# Self‐Leadership Based on Caring Among Primary Nurses: A Qualitative Study in Hospital Settings

**DOI:** 10.1155/jonm/5581421

**Published:** 2026-06-24

**Authors:** Mahmud Ady Yuwanto, Iyus Yosep, Iqbal Pramukti, Ati Surya Mediawati

**Affiliations:** ^1^ Doctoral Study Program in Medical Science, Faculty of Medicine, Universitas Padjadjaran, Bandung, Indonesia, unpad.ac.id; ^2^ Department of Fundamental Nursing, Faculty of Health Sciences, Universitas Dr. Soebandi, Jember, Indonesia; ^3^ Department of Community and Mental Health Nursing, Faculty of Nursing, Universitas Padjadjaran, Bandung, Indonesia, unpad.ac.id; ^4^ Department of Fundamental Nursing, Faculty of Nursing, Universitas Padjadjaran, Bandung, Indonesia, unpad.ac.id

**Keywords:** caring, nursing management, primary nursing, qualitative study, self-leadership

## Abstract

Healthcare organisations increasingly require nurses to enact leadership within everyday clinical practice to sustain care quality in complex and resource‐constrained environments. This study aimed to explore how self‐leadership grounded in caring is enacted by primary nurses and to examine organisational and relational factors shaping this process. A qualitative descriptive design was employed, involving semistructured interviews with ten primary nurses working across inpatient wards, intensive care units and emergency departments. Data were analysed using thematic analysis. Six interrelated themes were identified: self‐regulation as the foundation of self‐leadership, caring practices as a relational framework for care delivery, professional identity and clinical autonomy, systemic and interpersonal barriers, organisational support as an enabling condition and perceived impacts on care quality. The findings indicate that leadership enacted at the point of care operates through self‐regulation and caring‐oriented interactions, supporting professional accountability and autonomous decision‐making, while being shaped by organisational conditions and systemic constraints. Participants perceived that integrating leadership with caring may enhance care quality, strengthen nurse–patient relationships and support professional satisfaction. This study contributes to nursing management scholarship by providing empirical insight into leadership as a relational and contextually situated process in everyday care rather than solely an individual competency, suggesting the relevance of organisational strategies that support nurses’ leadership and caring practices.

## 1. Introduction

Healthcare organisations are facing increasing complexity due to rising patient acuity, workforce shortages, and heightened expectations for quality and safety [1, 2]. Within this environment, nurses particularly those functioning as primary nurses are required to assume substantial responsibility for coordinating care, making timely clinical decisions, and sustaining therapeutic relationships with patients and families [3]. These demands have intensified attention on leadership capacities that are enacted in daily clinical practice rather than confined to formal managerial roles [4]. For nursing management, this shift highlights the importance of leadership approaches that are distributed, context‐sensitive and embedded at the point of care.

Self‐leadership has emerged as a relevant framework for understanding how nurses regulate their own behaviour, cognition and motivation in complex work settings [5]. Defined as the process by which individuals influence themselves to achieve optimal performance [6], self‐leadership has been linked to enhanced job performance, work engagement, professional autonomy and reduced burnout among nurses [7, 8]. Unlike hierarchical leadership models, self‐leadership emphasises self‐regulation and responsibility, making it particularly salient for primary nurses who must exercise independent judgement while navigating dynamic clinical demands.

At the same time, caring remains the philosophical and relational foundation of nursing practice. Caring theories, such as Watson’s Theory of Human Caring and Swanson’s Caring Theory, conceptualise caring as an intentional, relational process that shapes nurses’ interactions with patients, families and colleagues [9, 10]. Empirical evidence indicates that caring‐oriented practices are associated with improved patient satisfaction, stronger therapeutic relationships and positive perceptions of care quality [11, 12]. From a nursing management perspective, caring also contributes to organisational culture, staff engagement and the sustainability of high‐quality care.

Despite their shared relevance, self‐leadership and caring have largely been examined as separate constructs within the literature [13]. Research on self‐leadership has tended to focus on cognitive and behavioural self‐regulation strategies, often within organisational psychology or management contexts, while caring scholarship has prioritised ethical and relational dimensions of nursing practice [14–16]. This separation limits understanding of how self‐leadership is enacted through caring in clinical environments, particularly in roles that demand both autonomy and sustained relational engagement. Addressing this gap is critical for nursing management, as it provides insight into how leadership processes operate at the intersection of individual agency, professional values and organisational expectations.

The primary nursing model offers an important context for examining this integration. Primary nursing emphasises continuity of care, accountability and nurse autonomy, positioning the primary nurse as the coordinator and leader of a patient’s care [17, 18]. While studies have reported positive outcomes associated with primary nursing, including enhanced accountability and patient satisfaction [19, 20], limited empirical attention has been given to the internal leadership processes that enable nurses to fulfil these responsibilities effectively. There is a lack of qualitative evidence exploring how self‐leadership grounded in caring values supports primary nurses in managing complex clinical and organisational demands.

From a nursing management standpoint, understanding self‐leadership based on caring is increasingly important. Healthcare organisations often rely on nurses’ capacity for self‐direction to maintain care quality under resource constraints; however, an exclusive focus on individual responsibility risks overlooking the relational and organisational conditions necessary for sustainable practice. Integrating self‐leadership with caring offers a more holistic perspective that aligns with contemporary leadership approaches emphasising relational leadership, professional empowerment, and shared accountability [21–23].

Despite growing interest in self‐leadership and caring within nursing scholarship, these constructs are often examined separately, with limited understanding of how self‐leadership is enacted through caring in everyday clinical management. Existing nursing leadership research has largely focused on formal roles or individual psychological outcomes, offering limited insight into leadership processes embedded in primary nursing practice. Addressing this gap, this study explores self‐leadership based on caring among primary nurses, conceptualising caring as a relational mechanism that enables self‐regulation, clinical autonomy and accountable decision‐making within complex organisational contexts.

Importantly, self‐leadership is not understood as an isolated individual capacity but as a contextually embedded process shaped by organisational conditions, relational interactions and systemic constraints.

## 2. Materials and Methods

### 2.1. Study Design

This study employed a qualitative descriptive design with a thematic analysis approach to explore how self‐leadership based on caring is enacted by primary nurses within hospital settings. A qualitative descriptive approach was considered appropriate to capture participants’ experiences and perspectives in a manner that remains close to the data while allowing systematic interpretation of patterns relevant to nursing management practice [24, 25].

Thematic analysis was used to identify recurring patterns of meaning related to self‐regulation, caring practices, professional roles, organisational support and perceived outcomes of self‐leadership. This approach enabled an examination of leadership processes as they are enacted in everyday clinical work, rather than as abstract or role‐based constructs, aligning with the study’s focus on leadership embedded in practice rather than formal managerial positions.

### 2.2. Study Setting and Context

The study was conducted in a hospital setting in Indonesia, involving multiple clinical units, including inpatient wards, intensive care units (ICU) and emergency departments (EDs). These settings were selected to capture diverse organisational and clinical contexts in which primary nurses are required to exercise self‐leadership, manage complex patient needs and coordinate care across professional boundaries. The hospital applies a primary nursing model, in which nurses assume accountability for planning, delivering and evaluating patient care within their scope of practice.

Like many hospital settings internationally, nursing practice in this context involves managing high clinical workloads and frequent interactions with patients’ families. These organisational conditions influence how nurses enact leadership and caring practices in daily clinical work, particularly in relation to prioritisation, communication and professional accountability.

### 2.3. Participants and Sampling

Participants were registered nurses working as primary nurses in the selected hospital. A purposive sampling strategy was employed to recruit participants who were able to provide rich and relevant insights into self‐leadership and caring practices within primary nursing roles. Inclusion criteria were as follows: (1) actively functioning as a primary nurse, (2) having direct responsibility for coordinating patient care and (3) willingness to participate in an in‐depth interview.

A total of ten primary nurses (R1–R10) participated in the study. The sample size was considered sufficient as data saturation was achieved. Data saturation was determined when no new substantive themes or variations in meaning emerged across successive interviews, and existing categories were sufficiently developed. Saturation was assessed concurrently with data collection, and interviews continued until thematic redundancy was observed across participants in the later interviews [26]. Participants represented a range of clinical units and professional experience levels, enhancing the breadth of perspectives captured and supporting the transferability of the findings.

### 2.4. Data Collection

Data were collected through individual semistructured interviews conducted during November 2025. A semistructured interview guide was developed based on self‐leadership theory (Manz and Neck) and caring theories (Watson and Swanson) and refined through expert consultation to ensure relevance to nursing management contexts. The guide included open‐ended questions exploring participants’ understandings of self‐leadership based on caring, experiences in clinical practice, perceived challenges, organisational support and perceived impacts on care delivery. The interview guide, including key questions and prompts used during data collection, is provided in Supporting File 1.

Interviews were conducted in a private setting within the hospital or at a mutually agreed location to ensure confidentiality and participant comfort. Each interview lasted approximately 45–60 min and was audio‐recorded with participants’ consent. Field notes were taken during and after interviews to capture contextual information and enhance analytic sensitivity.

### 2.5. Data Analysis

Data were analysed using Braun and Clarke [24] reflexive thematic analysis, adapted to a qualitative descriptive approach with emphasis on close adherence to the data and limited theoretical abstraction. Interview transcripts were read repeatedly to achieve data familiarisation, followed by the generation of initial codes representing meaningful units related to self‐leadership, caring practices, professional roles, organisational support and outcomes relevant to nursing management. Codes were iteratively compared and organised into subthemes, which were then refined into six final themes through collaborative discussion and ongoing reflection among the research team. Themes were reviewed against the full dataset to ensure coherence and relevance to nursing management practice. An audit trail was maintained throughout the analytic process to support transparency and rigour. Detailed coding frameworks, representative verbatim quotations and analytical matrices illustrating the progression from codes to subthemes and themes are provided in Supporting Files 1–3.

### 2.6. Rigour and Trustworthiness

Rigour and trustworthiness were ensured using the criteria of credibility, dependability, confirmability and transferability [27]. Credibility was supported through iterative engagement with the data and the use of representative verbatim quotations, while dependability and confirmability were enhanced by maintaining a transparent audit trail documenting analytic decisions. Transferability was facilitated by providing detailed descriptions of the study context and participants, and researcher reflexivity was further strengthened through ongoing reflection on assumptions and analytic interpretations throughout the study.

Coding and theme development were conducted collaboratively among members of the research team. Differences in coding interpretations were discussed until consensus was reached, ensuring consistency and analytical credibility. Peer debriefing was conducted within the research team to ensure alignment between data, codes and thematic interpretations. The research team consisted of nursing academics with expertise in nursing management and qualitative research, and reflexivity was maintained through continuous reflection on assumptions and interpretive positioning.

### 2.7. Ethical Considerations

This study was approved by the Research Ethics Committee of Universitas Padjadjaran (Approval No. 902/UN6.KEP/EC/2025). Written informed consent was obtained from all participants prior to data collection. Participants were assured of confidentiality, anonymity and their right to withdraw from the study at any time without consequence. All data were securely stored and accessible only to the research team.

## 3. Results

### 3.1. Participant Characteristics

This qualitative study involved ten registered nurses (R1–R10) who were working as primary nurses across various hospital units, including inpatient wards, ICU and ED. Participants had diverse clinical backgrounds and varying lengths of professional experience, enabling the capture of rich and heterogeneous perspectives on the implementation of self‐leadership based on caring in daily nursing practice. All participants were directly responsible for coordinating and delivering patient care and were held accountable for clinical decision‐making within their scope of practice.

### 3.2. Overview of Qualitative Findings

**FIGURE 1:**
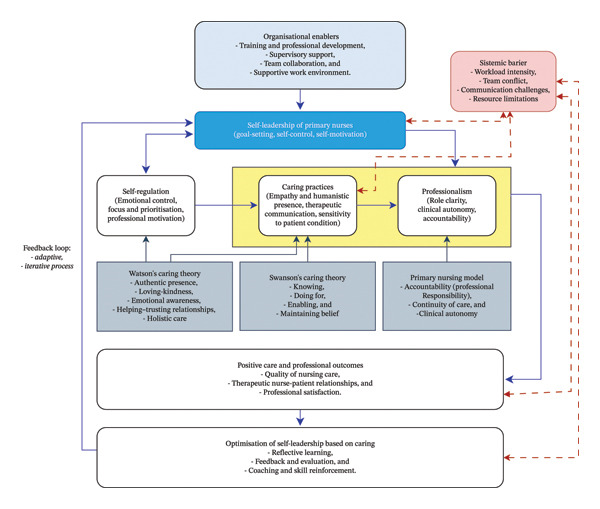
Conceptual model of self‐leadership based on caring among primary nurses.

Thematic analysis of the interview data resulted in six interrelated themes that collectively describe how self‐leadership based on caring is enacted by primary nurses in hospital settings. These themes are as follows: (1) Self‐regulation as the foundation of nurse self‐leadership, (2) Caring practices as a relational framework for nursing care, (3) Professional identity and clinical autonomy of primary nurses, (4) Systemic and interpersonal barriers to self‐leadership–based caring, (5) Organisational support as an enabling condition for self‐leadership and (6) Perceived impacts of self‐leadership–based caring on care quality. Six interrelated themes were identified from the analysis, describing how self‐leadership based on caring is enacted by primary nurses. The relationships among these themes are illustrated in Figure 1.

### 3.3. Theme 1: Self‐Regulation as the Foundation of Nurse Self‐Leadership

Additional verbatim quotations supporting each theme and subtheme are presented in Supporting File 2.

Participants consistently described self‐regulation as the core element underlying their capacity to lead themselves in clinical practice. Participants described these practices not only as clinical competencies but also as reflecting leadership‐related processes such as self‐direction, prioritisation, initiative and accountability in managing patient care.

Nurses highlighted the need to manage their emotions when encountering challenging patient situations or demanding family members. One participant stated, *“I have to restrain myself, stay patient, and remain calm so that the patient feels comfortable”* (R1). Similarly, another nurse emphasised maintaining composure amid workload pressure and family demands, noting that emotional stability was essential to preserve clinical focus (R8).

Prioritisation and decision‐making were also integral to self‐regulation. Participants described systematically assessing patient conditions and determining priorities, particularly in high‐acuity environments. For example, a nurse working in emergency care explained, *“I determine priorities using the ABCD approach and ensure that actions match the patient’s condition”* (R7). Another participant described initiating detailed observation and reassessing priorities when patient conditions deteriorated unexpectedly (R8).

Motivation and reflective practice further supported self‐regulation. Nurses frequently referred to recalling their professional purpose to sustain commitment and resilience. One participant noted, *“I remind myself why I became a nurse; even small actions can have a big impact on patients”* (R1). Reflection on prior actions was described as a mechanism for continuous improvement and self‐guidance in practice (R5).

### 3.4. Theme 2: Caring Practices as a Relational Framework for Nursing Care

Participants described caring as a central relational framework that shaped how nurses enacted self‐leadership in patient care. Caring practices were reflected through empathy, therapeutic communication and sensitivity to subtle clinical changes.

Empathy was repeatedly identified as fundamental to understanding patients holistically. Nurses described caring as the ability to “humanise” patients, even when patients were unconscious or unable to communicate. As one participant stated, *“Empathy allows me to see the patient as a human being, even when they are not conscious”* (R3). Another nurse emphasised emotional attunement as a guide for clinical responsiveness (R8).

Therapeutic communication enabled nurses to elicit patient concerns, provide reassurance and manage expectations. One participant explained, *“Through effective communication, I can understand what the patient truly needs”* (R2). Communication was also used strategically to reduce tension with patients and families, particularly in high‐pressure contexts such as the ED (R10).

Sensitivity to changes in patients’ condition was closely linked to caring. Participants described vigilance towards minor physiological or behavioural changes that could indicate clinical deterioration. A nurse working in critical care explained, *“I must be alert to even small changes, because they can be crucial for patient safety”* (R5). Another participant highlighted that caring heightened awareness and responsiveness during acute situations (R8).

### 3.5. Theme 3: Professional Identity and Clinical Autonomy of Primary Nurses

Participants described how self‐leadership based on caring was associated with reinforcing nurses’ professional identity and clinical autonomy. Participants articulated a strong sense of responsibility for patient outcomes and emphasised their role as primary decision‐makers within nursing care.

Participants perceived self‐leadership as integral to fulfilling their professional role. One nurse stated, *“As a primary nurse, I am fully responsible for making appropriate decisions for my patients”* (R2). Nurses emphasised that professional identity extended beyond executing medical orders and involved independent clinical judgement (R7).

Clinical autonomy was particularly evident in critical situations. Nurses described initiating nursing interventions within their scope of practice before consulting other professionals when necessary. As one participant noted, *“I take action according to my authority without waiting for instructions”* (R5). Another nurse described performing initial assessments and stabilisation measures prior to interdisciplinary collaboration (R8).

Accountability was closely associated with professionalism. Participants indicated that self‐leadership enabled more structured and deliberate actions. One nurse remarked, *“Self-leadership makes my actions more organised and prevents careless practice”* (R3). Nurses also associated caring with professional accountability, as it encouraged attentiveness to patient comfort, safety and dignity.

### 3.6. Theme 4: Systemic and Interpersonal Barriers to Self‐Leadership Based on Caring

Despite recognising the importance of self‐leadership based on caring, participants reported multiple barriers that constrained its consistent implementation. Participants described these barriers as relating to family‐related pressures, team‐related challenges and workload‐related constraints.

Family‐related barriers were frequently described. Nurses reported difficulties managing unrealistic expectations or negative perceptions from patients’ relatives. One participant explained, *“Families sometimes give negative opinions without understanding the actual condition”* (R1). Another noted that family pressure could reduce concentration and increase emotional strain (R8).

Interpersonal barriers within nursing teams also emerged. Participants reported inconsistent teamwork and lack of shared understanding regarding caring principles. A nurse stated, *“Sometimes colleagues do not align with the caring approach I try to apply”* (R2). Ineffective communication across units further complicated care coordination (R7).

Workload and staffing shortages were prominent systemic barriers. Nurses working in emergency and high‐acuity settings described patient overload as a major obstacle. One participant noted, *“When the patient load is excessive, I have to exert extra effort just to maintain emotional control”* (R10). High workload was perceived to limit opportunities for reflective practice and personalised caring interactions (R9).

### 3.7. Theme 5: Organisational Support as an Enabling Condition for Self‐Leadership

Participants consistently identified organisational support as an important enabling factor of self‐leadership based on caring. Support mechanisms included training opportunities, leadership support and effective teamwork structures.

Training and professional development were viewed as essential for strengthening both caring and leadership competencies. One participant stated, *“Continuous training in caring and leadership is necessary to build confidence”* (R1). Others emphasised the importance of equitable access to training, particularly for newly employed nurses (R8).

Leadership support played a significant role in reinforcing self‐leadership. Participants valued guidance, supervision and encouragement from nurse managers. As one nurse noted, *“Leadership support is crucial for providing direction and reassurance”* (R7).

Team collaboration further facilitated self‐leadership. Nurses described effective teamwork as enhancing responsiveness and reducing individual burden. One participant explained, *“Strong teamwork allows care to be delivered more efficiently”* (R4). Rapid interprofessional collaboration was also highlighted as important for managing complex cases (R8).

### 3.8. Theme 6: Perceived Impacts of Self‐Leadership–Based Caring on Care Quality

The final theme captures participants’ perceptions of the outcomes associated with self‐leadership based on caring. These outcomes were described as including perceived improvements in care quality, strengthened nurse–patient relationships and enhanced professional satisfaction.

Participants perceived that self‐leadership contributed to more focused and timely nursing care. One nurse stated, *“When I can regulate myself and understand the patient, communication improves and care becomes more effective”* (R1). Another noted that self‐leadership resulted in care that was more aligned with patient needs (R8).

Relationships with patients were also positively affected. Participants reported increased patient trust and comfort when caring practices were consistently applied. As one nurse described, *“Patients become more comfortable and trusting, and they often provide positive feedback”* (R6).

Professional satisfaction emerged as a meaningful outcome. Nurses expressed a sense of fulfilment when their actions led to observable patient improvement. One participant remarked, *“I feel satisfied and confident when I see that my actions have a positive impact on patients”* (R3). Self‐leadership was thus perceived as reinforcing both care quality and nurses’ sense of professional purpose.

This figure illustrates that self‐leadership based on caring is enacted as a dynamic and contextually embedded process in clinical practice. Primary nurses described how individual self‐regulation, relational caring practices and professional identity interact in their daily work, enabling them to exercise clinical autonomy, accountability and goal‐directed behaviour.

These processes are supported by organisational factors such as leadership support, training opportunities and collaborative work environments, while simultaneously being shaped by systemic constraints, including workload intensity, team‐related challenges and resource limitations. Nurses reported continuously adapting their self‐leadership practices through reflection and experiential learning in response to these contextual conditions.

Taken together, the findings describe how self‐leadership based on caring is enacted as a dynamic and contextually embedded process in clinical practice. Primary nurses described how individual self‐regulation, relational caring practices, and professional identity interact in their daily work, enabling them to exercise clinical autonomy, accountability, and goal‐directed behaviour.

These processes are supported by organisational factors such as leadership support, training opportunities and collaborative work environments, while simultaneously being shaped by systemic constraints, including workload intensity, team‐related challenges and resource limitations.

The analytical relationships between codes, subthemes and themes underpinning these findings are documented in Supporting File 3, which provides a code–subtheme–theme matrix supporting theme development. Together, these findings provide a descriptive account of how self‐leadership based on caring is enacted by primary nurses in complex clinical environments and form the basis for the following discussion.

## 4. Discussion

This study contributes to nursing management scholarship by demonstrating that self‐leadership among primary nurses is not solely an individual cognitive or behavioural capacity, but a relational and context‐dependent process enacted through caring practices within everyday clinical work. By empirically integrating self‐leadership theory with caring frameworks and the primary nursing model, this study provides empirical insight into how leadership is operationalised at the point of care. Specifically, caring is interpreted merely not only as an ethical or interpersonal value but also as a functional leadership mechanism that enables self‐regulation, professional autonomy and accountable decision‐making in complex organisational settings. Specifically, caring operates as a process through which self‐leadership is enacted by guiding nurses’ attention, shaping clinical priorities and informing decision‐making in real‐time clinical interactions.

Through an in‐depth qualitative exploration of primary nurses’ experiences, six interrelated themes were identified (Table 1), illustrating how self‐regulation, caring practices, and professional identity are dynamically shaped by organisational support and systemic constraints. Together, these findings contribute to the nursing leadership literature by highlighting the embedded nature of self‐leadership within relational, professional and organisational contexts, thereby providing a more comprehensive understanding of leadership processes relevant to contemporary nursing management.

**TABLE 1:** Themes and subthemes of self‐leadership based on caring among primary nurses.

Theme	Theme description	Subthemes
Theme 1. Self‐regulation as the foundation of nurse self‐leadership	Primary nurses described self‐regulation as a core capacity enabling them to manage emotions, maintain focus, prioritise care and sustain motivation when facing complex clinical situations.	a. Emotional control and composure under pressure
		b. Prioritisation and rapid clinical decision‐making
		c. Professional motivation and reflective practice

Theme 2. Caring practices as a relational framework for nursing care	Caring was perceived as the relational basis through which self‐leadership was enacted, guiding nurses’ empathy, communication and attentiveness to patient needs.	a. Empathy and humanistic presence
		b. Therapeutic communication with patients and families
		c. Sensitivity to subtle changes in patient condition

Theme 3. Professional identity and clinical autonomy of primary nurses	Self‐leadership strengthened nurses’ professional identity, characterised by accountability, autonomous decision‐making and responsibility for holistic patient care.	q. Understanding professional role and authority
		b. Clinical autonomy within nursing scope of practice
		c. Accountability and professional responsibility

Theme 4. Systemic and interpersonal barriers to self‐leadership–based caring	Participants identified barriers that constrained caring‐oriented self‐leadership, arising from interpersonal dynamics and organisational pressures.	Family‐related pressures and unrealistic expectations
		Team‐related challenges and coordination issues
		Workload intensity and staffing limitations

Theme 5. Organisational support as an enabling condition for self‐leadership	Organisational structures and leadership practices were viewed as essential enablers for sustaining self‐leadership based on caring.	a. Access to training and professional development
		b. Leadership support and supervision
		c. Team collaboration and interprofessional coordination

Theme 6. Perceived impacts of self‐leadership–based caring on care quality	Nurses perceived that integrating self‐leadership and caring resulted in improved care processes, stronger relationships and enhanced professional satisfaction.	a. Improved quality and focus of nursing care
		b. Strengthened nurse–patient relationships
		c. Increased professional satisfaction and confidence

Importantly, the six themes should be understood as interrelated rather than discrete components. These findings suggest that self‐leadership based on caring is not a linear process but a dynamic interaction between individual capabilities, relational practices and organisational contexts.

### 4.1. Self‐Regulation as a Core Mechanism of Nurse Self‐Leadership

The findings indicate that self‐regulation constitutes the foundational mechanism through which self‐leadership is exercised by primary nurses. Emotional control, prioritisation of care and sustained motivation emerged as essential capabilities enabling nurses to function effectively in high‐pressure environments. This aligns closely with the conceptualisation of self‐leadership proposed by Manz and Neck, which emphasises self‐regulatory strategies such as self‐observation, self‐goal setting and constructive thought patterns as drivers of effective self‐direction [28, 29].

In the context of nursing management, these findings reinforce prior evidence suggesting that nurses who demonstrate strong self‐regulation are better equipped to manage role stressors, maintain care quality and support team functioning [22, 30]. Importantly, the present study extends this understanding by illustrating that self‐regulation is frequently mobilised in response to relational demands, particularly interactions with patients’ families and multidisciplinary teams. Thus, self‐regulation in nursing should be understood not only as an internal competency but also as a managerial capacity that sustains professional conduct under organisational pressure.

### 4.2. Caring as a Relational Enabler of Self‐Leadership

Caring practices were found to function as a relational framework through which self‐leadership is translated into meaningful clinical action. Empathy, therapeutic communication and sensitivity to subtle patient changes were consistently described as guiding nurses’ decision‐making and behavioural responses [31, 32]. These findings resonate strongly with Watson’s Theory of Human Caring, which positions caring as a moral and relational foundation for professional nursing practice [10, 33]. The emphasis on the presence, empathy, and authentic engagement observed in this study reflects Watson’s carative factors, particularly the cultivation of helping trusting relationships.

Similarly, Swanson’s caring theory provides a useful lens for interpreting these findings. The processes of *knowing*, *being with* and *doing for* were evident in nurses’ descriptions of vigilant observation, emotional presence and proactive intervention [9, 34]. From a management perspective, these caring processes appear to strengthen nurses’ situational awareness and clinical judgement, thereby supporting effective self‐leadership.

The integration of caring and self‐leadership highlighted in this study contributes to nursing management scholarship by demonstrating that relational competence is not separate from leadership capability but rather a core mechanism through which leadership is enacted at the point of care.

### 4.3. Professional Identity and Clinical Autonomy in Primary Nursing

The third theme underscores the role of self‐leadership in reinforcing professional identity and clinical autonomy among primary nurses. Participants described self‐leadership as integral to assuming responsibility, exercising independent judgement and coordinating patient care within their scope of practice. These findings are consistent with the principles of the primary nursing model, which emphasises continuity of care, accountability and nurse autonomy as key organisational features [32, 35].

From a nursing management perspective, the findings suggest that self‐leadership enables nurses to operationalise professional autonomy in ways that align with organisational goals for quality and safety. Previous studies have linked nurse autonomy to improved patient outcomes and job satisfaction [36, 37]. The present study adds nuance by illustrating how autonomy is enacted through self‐leadership grounded in caring values, rather than through hierarchical authority or positional power.

### 4.4. Systemic and Interpersonal Barriers to Self‐Leadership–Based Caring

Despite strong internal capacities for self‐leadership, participants reported substantial systemic and interpersonal barriers that constrained their ability to consistently enact caring‐oriented leadership. Family pressures, team misalignment, communication delays and workload intensity were frequently cited challenges. These findings align with broader nursing management literature identifying workload, staffing shortages and interprofessional tensions as persistent threats to care quality and staff wellbeing [38–40].

Importantly, the data indicate that contextual barriers do not eliminate self‐leadership but instead intensify the cognitive and emotional demands experienced by nurses. This pattern aligns with theoretical perspectives that caution against framing self‐leadership as an individual‐level solution to structurally embedded organisational problems [41].

From an organisational standpoint, excessive reliance on individual self‐regulation in the absence of structural and managerial support may inadvertently increase the risk of burnout among nurses [42].

### 4.5. Organisational Support as a Critical Enabling Context

Consistent with nursing management theory, organisational support emerged as a decisive factor influencing the sustainability of self‐leadership based on caring. Participants emphasised the importance of equitable access to training, supportive supervision and effective teamwork. These findings are consistent with evidence that empowering work environments enhance nurses’ leadership behaviours, engagement and performance [43, 44].

Leadership support was perceived as legitimising nurses’ autonomous decision‐making while reinforcing caring‐oriented practice. These findings underscore the pivotal role of nurse managers in shaping organisational conditions that enable distributed and self‐directed leadership at the point of care [45, 46]. Investment in leadership development, structured reflective practice forums and interprofessional collaboration emerges as a strategic approach with the potential to enhance care quality and promote workforce sustainability.

### 4.6. Impacts on Care Quality and Professional Outcomes

Participants consistently described that the integration of self‐leadership and caring was associated with tangible improvements in care quality, reflected in more attentive clinical practice, strengthened nurse–patient relationships and heightened professional satisfaction. These perceived outcomes resonate with the existing literature demonstrating that relational leadership and caring‐oriented organisational cultures are positively associated with patient trust, safety and satisfaction, as well as nurses’ professional fulfilment [42, 47–49]. From an organisational perspective, these findings suggest that self‐leadership grounded in caring functions as a practice‐level mechanism through which everyday nursing work translates into sustained care quality and positive professional outcomes, particularly within complex healthcare settings.

### 4.7. Limitations

Several limitations should be acknowledged. This study was conducted within a single organisational context, which may limit transferability to other healthcare systems. Although rich qualitative data were obtained, the findings reflect participants’ perceptions and may be influenced by contextual and cultural factors. Additionally, responses may have been shaped by social desirability, particularly given the normative nature of caring in nursing practice. Future research could extend this work through multisite studies or mixed‐methods designs to examine the relationships between self‐leadership, caring and measurable organisational outcomes.

## 5. Implications for Nursing Management

The findings indicate that self‐leadership based on caring constitutes a critical managerial resource in contemporary nursing practice. Nursing managers should intentionally embed self‐leadership development within leadership and professional development programmes, with particular emphasis on self‐regulation, reflective learning and ethical decision‐making grounded in caring values. Creating supportive organisational environments through equitable access to training, consistent supervisory guidance, effective team communication and feedback mechanisms is essential to sustain nurses’ capacity for self‐directed and caring‐oriented practice. In addition, recognising and reinforcing the professional autonomy of primary nurses can strengthen accountability, enhance care quality and promote workforce engagement. Collectively, these strategies support organisational learning and continuous improvement, positioning nursing management to enhance both clinical performance and the relational foundations of high‐quality nursing care. Practically, nurse managers can implement structured reflective practice sessions, integrate self‐leadership modules into clinical training programmes and establish peer‐support systems to reinforce caring‐oriented leadership behaviours in daily practice.

## 6. Conclusion

This study provides empirical insight into how self‐leadership based on caring can be understood as a dynamic, relational and organisationally embedded process through which primary nurses enact leadership in everyday clinical practice. The findings suggest that self‐regulation, caring practices and professional autonomy can be understood as interconnected and mutually reinforcing processes shaped by organisational support and constrained by systemic conditions. By empirically integrating self‐leadership theory, caring frameworks and the primary nursing model, this study contributes to nursing management scholarship by highlighting caring as a functional leadership mechanism through which self‐leadership may be enacted in practice, rather than as a purely ethical ideal. The proposed conceptual model offers a potential framework for supporting leadership at the point of care through organisational support, reflective practice and system‐level approaches. Future research could further examine the applicability of this model across diverse healthcare settings and explore its implications for organisational performance and patient outcomes.

## Funding

The publication of this article was supported by Universitas Padjadjaran through the International Reputable Journal Publication Funding Program, under Grant No. 1719/UN6.RKT/Kep/HK/2025.

## Disclosure

The funding sources had no role in the study design, data collection, analysis, interpretation or manuscript preparation.

## Conflicts of Interest

The authors declare no conflicts of interest.

## Supporting Information

Additional supporting information can be found online in the Supporting Information section.

## Supporting information


**Supporting Information 1** Supporting File 1: Detailed coding framework and analytic process. This file provides the comprehensive open‐coding framework derived from qualitative interviews with primary nurses (RI–R10). It includes specific code labels (C1–C57), their detailed descriptions such as clinical prioritisation, empathy and professional accountability and the corresponding participant identifiers associated with each code.

**Supporting Information 2** Supporting File 2: Representative verbatim quotes by theme. This file presents a collection of direct quotations from the participants (primary nurses) that support the study’s findings. The quotes are organised according to the six main themes: Self‐regulation as the foundation of self‐leadership, Caring practices as a relational framework, Professional identity and clinical autonomy, Systemic and interpersonal barriers, Organisational support and The impacts of self‐leadership–based caring.

**Supporting Information 3** Supporting File 3: Code–subtheme–theme matrices supporting theme development. This file provides an analytical matrix that demonstrates the logical progression and relationship between the initial codes (C1–C57), the identified subthemes and the final six themes. It serves as a structural map showing how specific data points, such as emotional control and professional accountability, were synthesised into broader thematic categories such as self‐regulation and professional identity.

## Data Availability

Data sharing not applicable to this article as no datasets were generated or analysed during the current study.
