# Supplementary material for: Self‐Leadership Based on Caring Among Primary Nurses: A Qualitative Study in Hospital Settings
Source: J Nurs Manag. 2026 Jun 24;2026:5581421. doi: 10.1155/jonm/5581421 (PMC13291795; doi:10.1155/jonm/5581421)
Supplement: Supplementary file 1 — Supporting Information 1 Supporting File 1: Detailed coding framework and analytic process. This file provides the comprehensive open‐coding framework derived from qualitative interviews with primary nurses (RI–R10). It includes specific code labels (C1–C57), their detailed descriptions such as clinical prioritisation, empathy and professional accountability and the corresponding participant identifiers associated with each code. [file JONM-2026-5581421-s001.pdf]

Supplementary File 1: Detailed coding framework and analytic process

This supplementary file presents the detailed open coding derived from interviews with primary nurses (R1–R10), including code definitions and data sources.

Code	Code Description	Participant(s)
C1	Full physical, mental, and emotional presence during patient care	R1
C2	Building trust through greeting, attention, and listening	R1
C3	Patience in dealing with withdrawn or difficult patients	R1
C4	Recalling professional purpose to sustain motivation	R1
C5	Family-related pressures affecting nursing care	R1
C6	Need for caring and leadership training	R1
C7	Caring as the basis of professional responsibility	R2
C8	Effective communication to understand patient problems	R2
C9	Adapting to patients' cultural differences	R2
C10	Honesty and transparency in communication	R2
C11	Misalignment among nurses as a barrier	R2
C12	Team discussion and evaluation to enhance caring	R2
C13	Self-management through care planning	R3
C14	Maintaining focus and calm during crises	R3
C15	Empathy for unconscious patients	R3
C16	Heavy workload and complex coordination as barriers	R3
C17	Continuous learning through education and training	R3
C18	Self-leadership leading to structured care and reduced miscommunication	R3
C19	Caring as professional empathy in ICU settings	R4
C20	Emotional control when caring for critically ill patients	R4
C21	Careful attention to clinical changes in ICU	R4
C22	Delayed physician response as a barrier	R4
C23	Need for specialised training (ACLS, BHD)	R4
C24	Caring enhances patient comfort in ICU	R4
C25	Caring guiding rapid decision-making	R5
C26	Sensitivity to subtle patient changes	R5
C27	Non-cooperative colleagues as barriers	R5
C28	Professional work environment supporting self-leadership	R5
C29	Reflective practice after care actions	R5
C30	Therapeutic communication as the core of nurse–patient relationships	R6
C31	Managing family members who reject education	R6
C32	Collaboration with physicians and other professionals	R6
C33	Lack of peer support	R6
C34	Expectation for enhanced training to strengthen autonomy	R6
C35	Caring as viewing patients through nursing concepts	R7
C36	Clinical prioritisation using ABCD approach	R7
C37	Delayed inter-unit communication	R7
C38	External pressure disrupting continuity of care	R7
C39	Leadership support as a foundation of self-leadership	R7
C40	Nurses as professionals rather than task executors	R7
C41	Self-leadership as self-regulation and accountability	R8

Code	Code Description	Participant(s)
C42	Empathy and sensitivity as core caring values	R8
C43	Rapid observation and early action during deterioration	R8
C44	Motivation driven by patient safety	R8
C45	Family pressure and workload burden	R8
C46	Need for teamwork and equitable training	R8
C47	Self-leadership leading to timely and trusted care	R8
C48	Empathy and personal approach as caring	R9
C49	In-depth observation to identify patient complaints	R9
C50	Families demanding rapid care without understanding conditions	R9
C51	Need for equitable access to training	R9
C52	Excessive workload limiting optimal caring	R9
C53	Maintaining emotional stability during overload	R10
C54	Triage and prioritisation based on acuity	R10
C55	Education to reduce patient and family pressure	R10
C56	High patient volume in emergency settings	R10
C57	Self-leadership resulting in humane and low-conflict care	R10
