# Supplementary material for: Self‐Leadership Based on Caring Among Primary Nurses: A Qualitative Study in Hospital Settings
Source: J Nurs Manag. 2026 Jun 24;2026:5581421. doi: 10.1155/jonm/5581421 (PMC13291795; doi:10.1155/jonm/5581421)
Supplement: Supplementary file 2 — Supporting Information 2 Supporting File 2: Representative verbatim quotes by theme. This file presents a collection of direct quotations from the participants (primary nurses) that support the study’s findings. The quotes are organised according to the six main themes: Self‐regulation as the foundation of self‐leadership, Caring practices as a relational framework, Professional identity and clinical autonomy, Systemic and interpersonal barriers, Organisational support and The impacts of self‐leadership–based caring. [file JONM-2026-5581421-s002.pdf]

Supplementary File 2: Representative verbatim quotes by theme

This file provides representative verbatim quotations supporting each theme and subtheme identified in the study.

Theme	Subtheme / Focus	Representative Verbatim Quote	Participant
**Theme 1. Self-Regulation as the Foundation of Nurse Self-Leadership**	Emotional control and composure	"I have to restrain myself, stay patient, and remain calm so that the patient feels comfortable."	R1
	Prioritisation and adaptive decision-making	"I observe carefully, set priorities, and evaluate actions, especially when conditions change suddenly."	R8
**Theme 2. Caring Practices as a Relational Framework**	Empathy and humanistic presence	"Empathy allows me to humanise the patient, even when they are unconscious."	R3
	Sensitivity to patient condition	"Caring makes me more sensitive to small changes that can determine patient safety."	R8
**Theme 3. Professional Identity and Clinical Autonomy**	Professional responsibility	"As a primary nurse, I am fully responsible for making appropriate decisions."	R2
	Clinical autonomy	"I take initial action before collaborating with the physician when the patient's condition worsens."	R8
**Theme 4 Systemic and Interpersonal Barriers**	Family-related barriers	"Families sometimes give negative opinions without understanding the real condition."	R1
	Workload-related barriers	"High workload often reduces my focus."	R8
**Theme 5. Organisational Support as an Enabler**	Training and development	"Continuous training in caring and leadership is essential."	R1
	Team collaboration	"Strong teamwork and quick collaboration are very helpful."	R8
**Theme 6. Impacts of Self-Leadership-Based Caring**	Quality and humanity of care	"When I can lead myself, my care becomes more focused and humane."	R10
	Nurse–patient relationship	"Patients become more comfortable and trusting."	R6
