# Supplementary material for: Self‐Leadership Based on Caring Among Primary Nurses: A Qualitative Study in Hospital Settings
Source: J Nurs Manag. 2026 Jun 24;2026:5581421. doi: 10.1155/jonm/5581421 (PMC13291795; doi:10.1155/jonm/5581421)
Supplement: Supplementary file 3 — Supporting Information 3 Supporting File 3: Code–subtheme–theme matrices supporting theme development. This file provides an analytical matrix that demonstrates the logical progression and relationship between the initial codes (C1–C57), the identified subthemes and the final six themes. It serves as a structural map showing how specific data points, such as emotional control and professional accountability, were synthesised into broader thematic categories such as self‐regulation and professional identity. [file JONM-2026-5581421-s003.pdf]

**Supplementary File 3: Code–subtheme–theme matrices supporting theme development**

Analytical matrix demonstrating the relationship between codes, subthemes, and themes.

| Theme | Subthemes | Associated Codes |
|---|---|---|
| Theme 1<br>Self-regulation | Emotional control; prioritisation; motivation | C1, C3, C4, C13, C14, C20, C31, C41, C44, C53 |
| Theme 2.<br>Caring practices | Empathy; communication; sensitivity | C2, C8, C15, C19, C26, C30, C42, C48, C49, C55 |
| Theme 3.<br>Professional identity | Role clarity; autonomy; accountability | C7, C25, C35, C40, C41, C43, C47 |
| Theme 4.<br>Barriers | Family pressure; team issues; workload | C5, C11, C16, C22, C27, C33, C37, C45, C50, C52, C56 |
| Theme 5.<br>Organisational support | Training; leadership; collaboration | C6, C12, C23, C28, C34, C39, C46, C51 |
| Theme 6.<br>Impacts on care quality | Care quality; relationships; satisfaction | C18, C24, C47, C57 |
